# The Promising Therapeutic and Preventive Properties of Anthocyanidins/Anthocyanins on Prostate Cancer

**DOI:** 10.3390/cells11071070

**Published:** 2022-03-22

**Authors:** Javad Mottaghipisheh, Amir Hossein Doustimotlagh, Cambyz Irajie, Nader Tanideh, Alireza Barzegar, Aida Iraji

**Affiliations:** 1 Center for Molecular Biosciences (CMBI), Institute of Pharmacy/Pharmacognosy, University of Innsbruck, Innrain 80-82, 6020 Innsbruck, Austria; 2 Medicinal Plants Research Center, Yasuj University of Medical Sciences, Yasuj 75918-67319, Iran; amirhosseindoustimotlagh@gmail.com; 3 Department of Clinical Biochemistry, Faculty of Medicine, Yasuj University of Medical Sciences, Yasuj 75918-67319, Iran; 4 Department of Medical Biotechnology, School of Advanced Medical Sciences and Technologies, Shiraz University of Medical Sciences, Shiraz 71348-14336, Iran; irajie@sums.ac.ir; 5 Stem Cells Technology Research Center, Shiraz University of Medical Sciences, Shiraz 71348-14336, Iran; tanidehn@gmail.com; 6 Pharmaceutical Sciences Research Center, Shiraz University of Medical Sciences, Shiraz 71348-14336, Iran; barzegarar@sums.ac.ir; 7 Liosa Pharmed Parseh Company, Shiraz 71997-47118, Iran; 8 Central Research Laboratory, Shiraz University of Medical Sciences, Shiraz 71348-14336, Iran

**Keywords:** prostate cancer, anthocyanidins, anthocyanins, cell cycle, cytotoxicity, apoptosis

## Abstract

As water-soluble flavonoid derivatives, anthocyanidins and anthocyanins are the plants pigments mostly rich in berries, pomegranate, grapes, and dark color fruits. Many bioactivity properties of these advantageous phytochemicals have been reported; among them, their significant abilities in the suppression of tumor cells are of the promising therapeutic features, which have recently attracted great attention. The prostate malignancy, is considered the 2nd fatal and the most distributed cancer type in men worldwide. The present study was designated to gather the preclinical and clinical studies evaluating potencies of anthocyanidins/anthocyanins for the treatment and prevention of this cancer type for the first time. In general, findings confirm that the anthocyanins (especifically cyanidin-3-O-glucoside) indicated higher activity against prostatic neoplasms compared to their correlated anthocyanidins (e.g., delphinidin); in which potent anti-inflammatory, apoptosis, and anti-proliferative activities were analyzed. Complementary anti-prostate cancer assessment of diverse naturally occurred anthocyanidins/anthocyanins and their synthetically optimized derivatives through preclinical experiments and eventually confirmed by clinical trials can promisingly lead to discover natural-based chemotherapeutic drug options.

## 1. Introduction

Prostate cancer (PC) is known as the 4th commonly diagnosed cancer (7.3% of total cases) in human and the 2nd mortal cancer type in men; moreover, in accordance with the global cancer burden (GLOBOCAN 2020), PC is reported as the most frequent cancer in men prevailing in 112 countries [1].

In the recent years, some systemic chemicals have been developed for the treatment of metastatic castration-resistant PC. Besides mitoxantrone, as the first cytotoxic chemotherapy approved by the US Food and Drug Administration (FDA), cabazitaxel, abiraterone acetate, enzalutamide, pembrolizumab, sipuleucel-T, as well as radioisotopes (e.g., samarium-153 and strontium-89) have been developed as successful PC therapeutic options [2]. Although chemotherapeutic drugs with possible side effects can suppress several tumors types, nature, the major source of diverse natural products, has always been inspiring for discovering healing pathways towards the human ailments. Several plant secondary metabolites (phytochemicals) and their semi-synthetically derivatives have been approved and utilized as anticancer drugs or adjuvant of chemical medications; paclitaxel (syn. taxol), vinblastine, vincristine, camptothecin, and etoposide are the most effective natural-based drugs which widely prescribed for the treatment of various cancer types [3,4,5,6,7].

Anthocyanidins (ACDs) and anthocyanins (ACNs), as the plant pigments are rich in many fruits and vegetables coloring red, violet, and blue, whilst mainly characterized as unsaturated, water-soluble, and unoxidized flavonoid derivatives. Potential anti-tumor capacities of these compounds have previously been reported [8], whereas they might be caused due to various related bioactivity properties of these compounds comprising anti-inflammation, antioxidant, anti-mutagenesis, anti-proliferation, stimulating autophagy or apoptosis, anti-invasion, anti-metastasis, etc. [9].

Although, some review articles focused on the pharmacological activities of anthocyanins [10,11,12,13], due to the increasing number and mortality rate of PC and anti-tumor potential of ACDs/ACNs, the present review was aimed at overviewing anticancer properties of these compounds experimented through preclinical investigations. The publications indexed in databases “PubMed, SciFinder, and Web of Science”, via searching the keywords “anthocyanidin” or “anthocyanin” plus “prostate cancer” have been targeted (Last search: 1 December 2021).

## 2. Chemistry of Anthocyanidins and Anthocyanins

**Figure 1:**
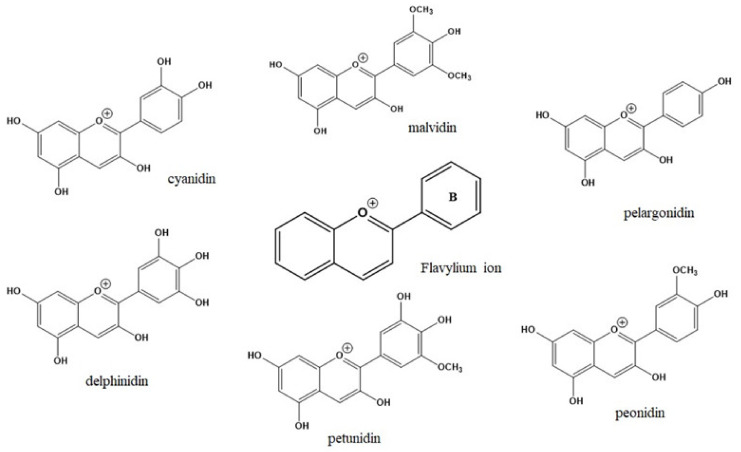
The chemical structures of flavylium ion and its derivatives known as anthocyanidins.

Flavylium ion (2-phenylchromenylium) with formula C_15_H_11_O^+^ and a molecular weight of 207.24724 g/mol is the basic structure of ACDs [14]. These aglycone compounds can be combined with sugar counterparts including glucose, galactose, and rhamnose, leading to biosynthesizing ACNs, in which the sugar part is attached to the hydroxyl group of pyran ring. As shown in Figure 1, the naturally identified ACDs and ACNs are derivatives of the main subclasses cyanidin, pelargonidin, peonidin, delphinidin, malvidin, and petunidin, whereas the substituents are mainly alter in the ring B [9].

### Chemical Stability and Bioavailability of Anthocyanidins/Anthocyanins

Taking into account that some review papers comprehensively overviewed and discussed the chemical stability and bioavailability of ACDs/ACNs [11,15,16,17], therefore in this section we confine to briefly conclude the information presented.

According to the chemical attributes of these natural compounds, they are mainly colored in pH under 3.5 in solutions, however, due to the copigmentation phenomena in nature, they can be detectable in higher pH values as well. It has been reported that ACNs are able to make noncovalent interactions with colorless copigments, subsequently this procedure can trigger formation of novel derivatives [15]. Furthermore, ACNs, via heating up, can easily be transformed to the colorless chalcone and carbinol derivatives, therefore a paler color can be observed [15].

After consumption, the gastrointestinal tract absorps ACDs/ACNs, however, the highest absorption and metabolism occurs in the distal lower bowel. The absorption will be continued in the intestine via microbial catabolism, whereas the combination of microbial–human metabolites evolves the absorption, and enhances bioavailability of these compounds [11]. Concerning the bioavailibity, it can be noted that its variability can be developed in three steps including food matrix and processing, responsible enzymes for metabolism and transmissions, and catabolizing gut microbiota [17].

## 3. Natural Sources of Anthocyanidins/Anthocyanins

Although the main natural sources of ACDs/ACNs are fruits, some cereal grains, legumes, and vegetables can further be considered as rich suppliers of these compounds. Bilberry (*Vaccinium myrtillus* L.), blueberry (*V. corymbosum* L. and *V. angustifolium* Ait.), blackberry (*Rubus* spp.), cranberry (*V. oxycoccus* L. and *V. macrocarpon* Ait.), saskatoon berry (*Amelanchier alnifolia* Nutt.), mulberry (*Morus alba* L.), haskap berry (*Lonicera caerulea* L.), and strawberry (*Fragaria x ananassa* Duch.) among the berries, besides apple (*Malus pumila* L.), orange (*Citrus sinensis* L.), pear (*Pyrus* spp.), apricot (*Prunus armeniaca* L.), red- (*Ribes rubrum* L.) and blackcurrant (*R. nigrum* L.), grape (*Vitis vinifera* L.), plum (*Prunus domestica* L.), cherry (*Prunus avium* L.), peach (*Prunus persica* L.), pomegranate (*Punica granatum* L.), and *Rosa* species have been reported the predominant fruit sources. Furthermore, followings are also known for their ACDs/ACNs content: *Asparagus officinalis* L., bean: *Phaseolus* spp., cabbage and cauliflower (*Brassica* spp.), carrot (*Daucus carota* L.), eggplant (*Solanum melongena* L.), ginger (*Zingiber officinale*), lentil (*Lens culinaris* Medic.), pea (*Pisum sativum* L.), peanut (*Arachis hypogaea* L.), pepper (*Capsicum annuum* L.), Potato (*Solanum tuberosum* L.), onion (*Allium cepa* L.), radish (*Raphanus sativus* L.), Rhubarb (*Rheum* spp.), soybean (*Glycine max* (L.) Merr.), sweet potato (*Ipomoea batatas* L.), Turnip (*Brassica campestris* L.). Moreover, some well-known cereals contain these compounds (barely, corn, rice, rye, sorghum, and wheat) [14,18,19].

## 4. Anti-Prostate Cancer Properties of Anthocyanidins/Anthocyanins

### 4.1. Metabolic Pathways of Prostate Cancer

**Figure 2:**
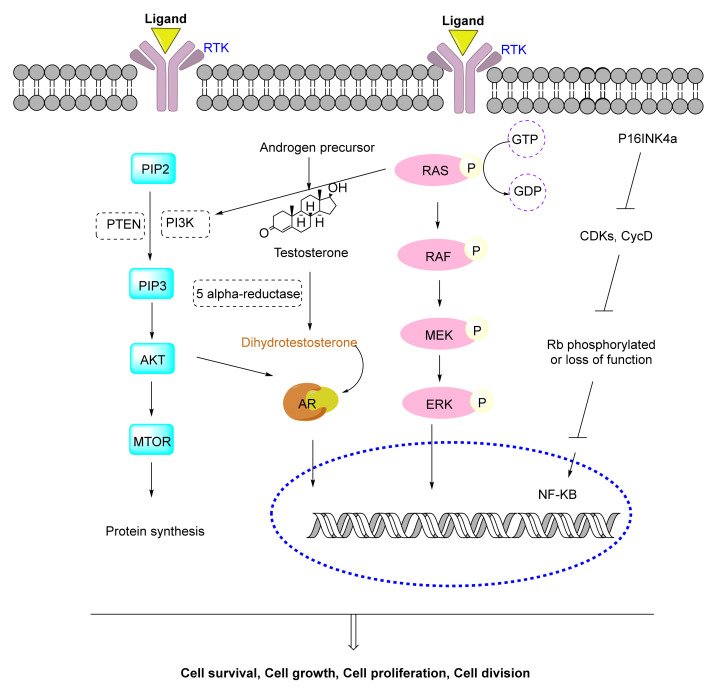
Schematic of some of the molecular pathways related to prostate cancer.

The cellular origin of PC is not very clear. However, it was shown that PC develops from normal prostate epithelium through multistep histological transformation processes, under molecular changes. The major signaling pathways that are most frequently altered in prostate cancer include androgen receptor (AR), the PI3K, Ras/Raf/MEK/ERK; and the retinoblastoma (RB) signaling pathway (Figure 2).

AR regulates the normal growth and development of the prostate gland. However, in PC, AR is overexpressed to some degree resulting in the serum PSA rising. AR signaling pathway in PC occurs via various mechanisms, including mutation or amplification of AR, the presence of intratumoral testosterone production, and the presence of splice variants of the AR receptor. Briefly, the binding of dihydrotestosterone leads to AR homodimerization, translocation to the nucleus, and recruitment to the androgen response elements (AREs) to initiate transcription [20,21,22].

The PI3K/Akt signaling pathway is part of a complex intracellular cell signaling cascade that regulates cellular metabolism, tumor development, growth, proliferation, metastases, and cytoskeletal reorganization. The PI3K/Akt pathway is initiated through the downstream of receptor tyrosine kinases (RTKs) activation. Next, PI3K catalyzes the phosphorylation of PIP2 to produce PIP3. Generation of PIP3 recruits PDK1 and Akt to the plasma membrane, resulting in subsequent phosphorylation of Akt by PDK1. Phosphorylated Akt starts a wide range of pathways but most importantly activates the mammalian target of rapamycin (mTOR), which is a serine/threonine kinase that plays a critical role in tumorigenesis, regulation of cell growth, survival, and division. Interaction between Akt and AR can lead to AR activation in a ligand-independent manner (androgen-independent manner), ultimately up-regulating genes involved in CRPC tumorigenesis. Also, the activated Akt regulates downstream effectors such as BAD, GSK3β, TSC2, and PRAS40 to control various cellular processes, including protein synthesis, cell proliferation, growth, and survial [23,24,25].

The extracellular molecules such as growth factors, hormones, tumor-promoting substances, and differentiation factors, bind to receptor tyrosine kinases (RTKs) and initiate the intracellular signals for regulating cell proliferation, differentiation, and survival through mitogen-activated protein kinase (MAPK) cascade. RAS proteins are placed on the cytoplasmic side of the cell membrane which is activated in GTP-bound conformation and inactivated in GDP-bound conformation resulting in the accumulation of active RAS proteins in cells followed by stimulation of RAF. RAS causes persistent activation of the ERK-MAPK cascade in human cancers. Activated ERKs translocate to the nucleus, where they phosphorylate and regulate various transcription factors, leading to change in gene expression [26,27].

The retinoblastoma (Rb) tumor suppressor protein plays a role in the control of the cell cycle, terminal differentiation, control the DNA damage response and promotes cell cycle arrest in G1. Its function is reverted by Rb phosphorylation by cyclin D-CDK, which inactivates Rb and promotes E2F1-mediated transcription [28,29,30]. This situation causes constitutive activation and the induction of uncontrolled cell proliferation.

Chemopreventive effect is stated as potency of natural, synthetic, or biological compounds to hinder cancer incidence. These substances are able to protect DNA damage, specifically by reversing or blocking proliferation of these nonmalignant cells [31]. In the clinical aspect, dietary phytochemicals and non-steroidal anti-inflammatory drugs (NSAID) can be consumed as chemopreventive agents in the populations with high cancer risk and the normal populations [32].

**Figure 3:**
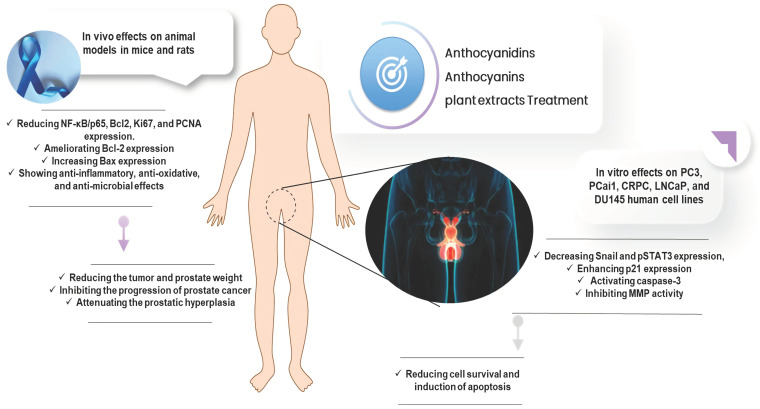
The mechanism of actions of anthocyanidins/anthocyanins on the treatment of prostate cancer.

The following sections provide evidence describing effects of the plant extracts rich in these phenolic compounds as well as the pure forms on the treatment and prevention of prostate cancer cells, which have been evaluated through various preclinical (in vitro, in vivo) studies. Figure 3 renders a general information concerning the mechanism of action of ACDs/ACNs and plant extracts towards prostate cancer cells.

### 4.2. In Vitro Screening of Anthocyanidins/Anthocyanins on Prostate Cancer

#### 4.2.1. Rich Anthocyanidins/Anthocyanins Plant Extracts

As documented in Table 1, most of the anticancer assessments of ACDs/ACNs in pure form and the enriched plant samples have been conducted in vitro against PC3, PCai1, CRPC (castration resistant cancer), LNCaP (androgen-independent prostate cancer), and DU145 (androgen-resistant tumoral prostatic) human cell lines. Among different evaluated plants, cranberry, jaboticaba peel, strawberry and wild blueberry (35% ethanol fraction) revealed higher capacities to target cancerous cell lines in order to reduce cell survival and induction of apoptosis compared to the rest of the tested extracts. Overal, the proposed mechanism of actions might involve down-regulation of cyclin-dependent kinase, enhanced expression of p21, activation of caspase-3, inhibition of MMP (matrix metalloproteinase) activity, and lessening Snail and pSTAT3 (phosphorylated-Signal transducer and activator of transcription-3) expression.

##### Rice

The potential activities of the methanolic and dichloromethane extracts of three purple rice (*Oryza sativa*) in Thailand, named Doisaket, Nan, and Payao combined with chemotherapeutic drugs on human cancer cells have previously been investigated. Methanolic extract of Payao was the most potent cytotoxic agent, via inhibition of LNCaP cell growth. Taking into account that through a quantitative analysis by utilization of HPLC, the methanolic extract of Payao indicated the highest ACN content (5.80 mg/g), thus these compounds are supposed to be the main bioactive constitiuents [33].

In another study, anti-proliferation action of the plant extracts of *Opuntia ficus Indica* flower and fruit of *Oryza sativa* (black rice) and their synergistic action on BPH-1 cells (epithelial cells of benign prostatic hypertrophy), LNCaP cells (androgen-dependent tumoral prostatic cells), and DU145 cells (androgen-resistant tumoral prostatic cells) were investigated. The flower extract of *O. ficus Indica* (10 µg/mL) and the extract of black rice (100 µg/mL) were individually capable to induce the reduction of cellular vitality of the BPH-1, LNCaP, and DU145 cell lines with the geometric mean of 70% and 80% cell viability, respectively. The treatment of the same cell lines with the combination of two extracts (210 µg/mL) could significantly lessen the cellular vitality (geometric mean of ~36%) with respect to the single extracts confirming a synergistic anti-proliferative effect [59].

**Figure 4:**
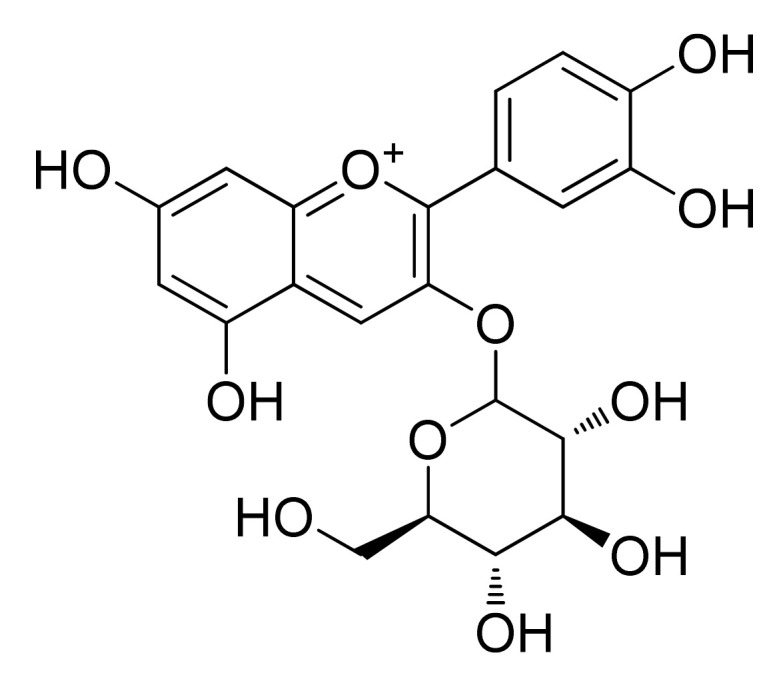
Chemical structure of cyanidin 3-O-glucoside.

Previously, the rice bran fraction enriched in ACNs was separated and processed until the major compound was acquired. The sub-fraction of the rice bran and the isolate cyanidin 3-O-glucoside (C3G) (Figure 4) were studied against PC3 using immunohistochemical-staining and immuno-blotting approaches. Both the extract and purified compound displayed cell viability with an IC_50_ value of 168 µM on PC3 cells. C3G reduced the expression of Smad/Snail signaling molecules at 100 µM and enhanced the expression of E-cadherin as cell surface protein by reducing the tumor transformation. This ACN was also able to inhibit MMP-9, in addition to reduction the nuclear factor kappa B (NF-κB) expression levels and the activity of the enzyme in PC3 cells leading to decelerate cell migration and invasion process [47].

In another study, insoluble fraction (1% hexane) of the purple rice ethanolic extract on transgenic rats against adenocarcinoma of the prostate showed lessening effect of adenocarcinoma in the lateral lobes of the prostate as well as prostatic intraepithelial neoplasia. Also, such diet significantly suppressed the tumor growth in PCai1 cells (castration-resistant prostate cancer xenograft model). In vitro evaluations on LNCaP and PCai1 cells recorded the suppression of the cell proliferation and induction of G0/G1 cell-cycle arrest. Interestingly, the cyclin D1, cdk4, androgen receptor (AR), and fatty acid synthase expression were down-regulated after treatment while expression of p38 mitogen-activated protein kinase and activation of AMP-activated protein kinase-α occurred in the treated PC cells, rat prostate tissues, and CRPC (Castration-resistant prostate cancer) tumors. Noteworthy, the chemical profile analysis of the extract characterized C3G as the active compound; subsequently the abovementioned potencies can be mainly correlated to this ACN [60].

##### Grape

Grape (*Vitis vinifera* L.) is a natural source of phenolic compounds specifically stilbenes and flavonoid derivatives including ACDs. Many bioactivities have been reported for this fruit, whereas the following properties can be highlighted: antioxidant, antimicrobial, cardio-, hepato-, neuro-, and skin-protective effects, as well as anti-tumor activities [61]. Few studies evaluated the preventive/treatment effects of this ACD-rich fruit on PC.

**Figure 5:**
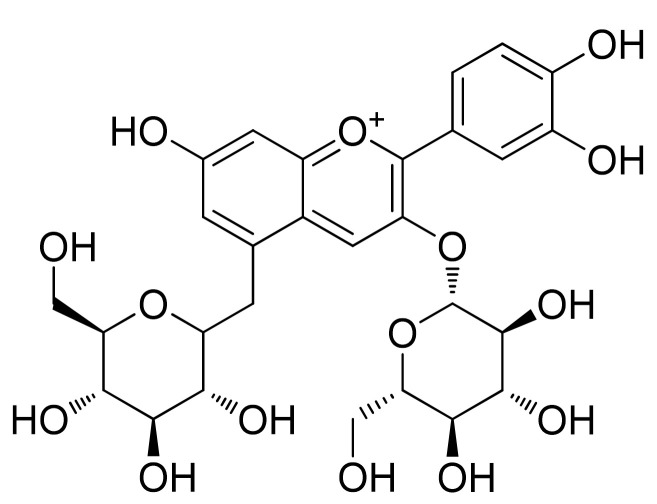
Chemical structure of cyanidin 3,5-diglucoside isolated from grape.

Burton et al. assessed the effects of muscadine grape skin extract containing ACNs as the main bioactive components on the prostate (LNCaP, ARCaP-E) and breast (MCF-7) cancer cells overexpressing snail transcription factor. It was documented that the snail overexpression led to increase cathepsin L expression/activity and pSTAT-3 which increased the invasion and migration. Muscadine grape skin extract at 5 μg/mL and 20 μg/mL inhibited snail overexpression leading to decrease cell invasion and migration. Phytochemical analysis showed cyanidin 3,5-diglucoside (Figure 5), ellagic acid, and ellagic acid precursors presented in the extract; however, the responsible bioactive compound has not been determined [34].

##### Cranberry

The tremendous health beneficial impacts of cranberry (*Vaccinium macrocarpon*) has been correlated to its phenolic contents such as flavonoids, proanthocyanidins, ACDs, and ACNs [62].

In a study performed by Déziel et al., the cell cycle arrest effect of American cranberry was confirmed by modulating the expression of cell cycle regulators. The treatment of DU145 with the cranberry extract at 25 and 50 mg/mL for 6 h decreased the proportion of cells in the G2/M phase and increased the proportion of cells in the G1 phase. Molecular analysis showed that the extract decreased the expression of CDK4, cyclin A, cyclin B1, cyclin D1, and cyclin E, in addition, increased the expression of p27. Moreover, the decrease in p16INK4a and an increase in pRBp107 protein expression levels were evident, although the changes were not statistically significant compared to the control [37].

In another study, 200 µg/mL of total cranberry extract and all fractions including sugars-, organic acids-, and polyphenols-enriched extract were evaluated against prostate (RWPE-1, RWPE-2, 22Rv1) cancer cell lines. The crude extract and all fractions represented at least 50% anti-proliferative activity against prostate cancer cells except the sugars-rich fraction [56], confirming the high potency of natural acids and polyphenols including ACDs/ACNs in the extract.

##### *Rhoeo discolor* 

The antiproliferative potency of 10 different crude extracts of *Rhoeo discolor* as well as the most bioactive subfractions have been tested against PC3 prostate cancer cell line, as well as a control fibroblast cell line NIH3T3. Results showed that mainly aqueous, methanolic, and ethanolic extracts were the most effective ones. Ten µg/mL of the crude extracts and 50 µg/mL of the experimented fractions significantly inhibited proliferation up to 61.8% in PC3. These natural compounds showed no significant ability to induce damage to the control cell line with less than around 28% apoptosis induction [63].

##### Maize

**Figure 6:**
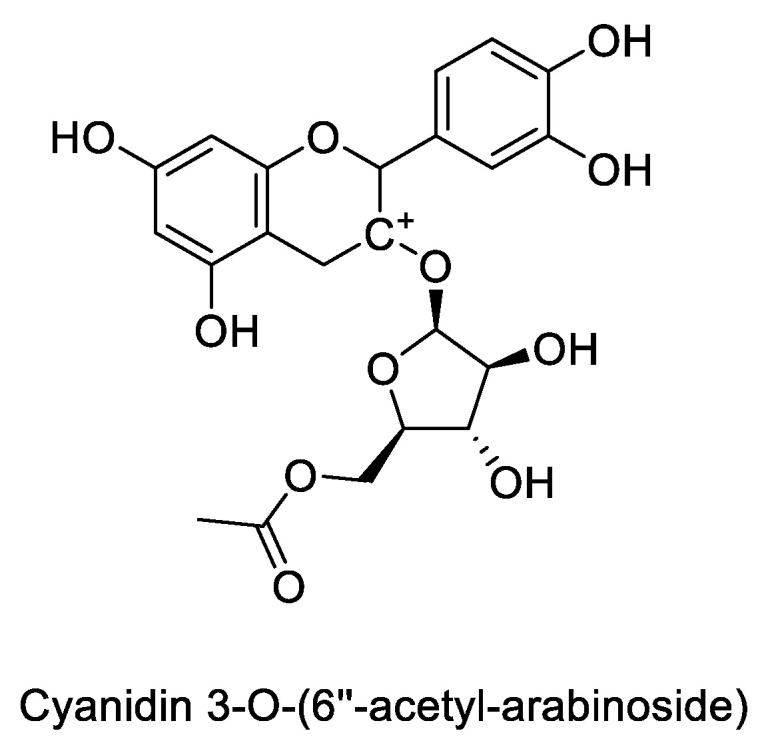
Chemical structure of cyanidin-3-O-(6′’-acetyl-arabinoside), one the major compounds of red maize.

Several studies reported red maize (*Zea mays* L.) as a valuable source of water-soluble pigments such as flavonoids and ACNs [64]. In a previous study, the chemical profile assessment of ACNs via HPLC-ESI-MS identified 20 compounds, characterizing cyanidin 3-p-hydroxy-benzoyl sophoroside-5-glucoside, C3G (Figure 4), and cyanidin-3-O-(6′-acetyl-arabinoside) (Figure 6) as major compounds. MTT assessments exerted significant antiproliferative activity and apoptosis effect in cell lines DU145 at 1000 μg/mL [65].

**Figure 7:**
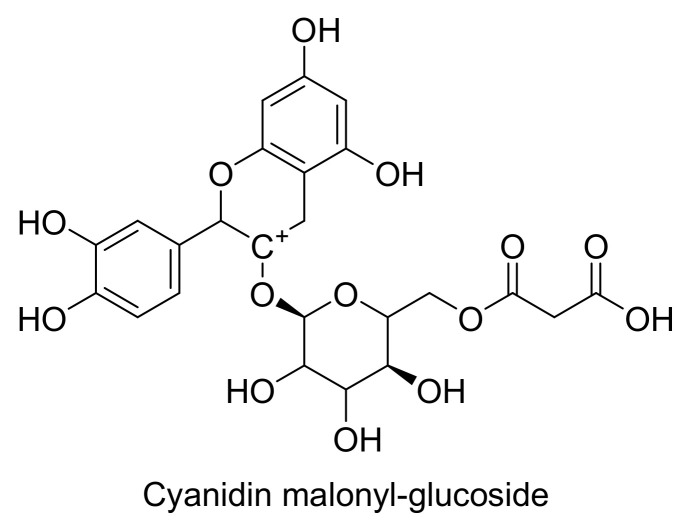
The most potent antiproliferative agent identified in blue maize.

Antiproliferative effects of blue maize as a natural source of phenolic acids and ACNs were evaluated against PC3 cancer cell. HPLC analysis showed ferulic acid and C3G (Figure 4) as the major phenolic and the most abundant anthocyanin compound, respectively. Among four different tested extracts, the acetone extract of native blue maize with just 30% cell viability was the most cytotoxic sample. In comparison, among the ACN compounds, cyanidin malonyl-glucoside (Figure 7) showed the best antiproliferative effects with the strongest correlation with the reduction of cell viability [41].

Preparative column chromatography was formerly used to purify ACNs from blue corn and tortilla. Results revealed that both of the ACNs rich extracts especially the tortillas extract was able to decrease the LNCaP cell viability and arrest the cell cycle in the G1 phase at 500 to 1000 µg/mL. Further assessments exhibited induction of apoptosis behavior on LNCaP cells when the ACN rich extract of tortilla was co-treated with 2-amino-N-quinolin-8-yl-benzenesulfonamide [66].

**Figure 8:**
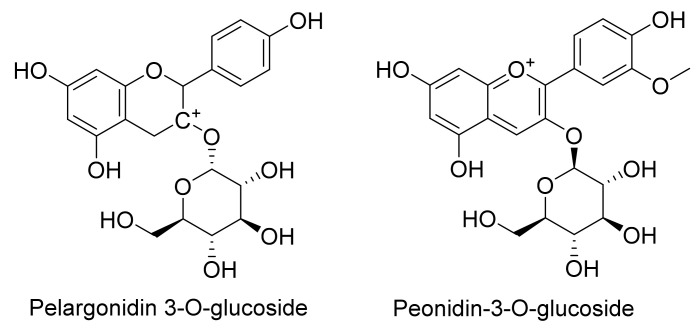
The main compounds found in purple corn.

Purple maize at 100 mg/L was able to inhibit the proliferation of LNCaP cells by decreasing the expression of cyclin D1 and inhibiting the G1 stage of the cell cycle. Eight weeks diet with 10% purple corn decreased the incidence of adenocarcinoma in the lateral prostate and slowed down the progression of PC through decrease the expression of cyclin D1, and downregulation of the Erk1 ⁄2 and p38 MAPK activation. Search for active compounds in purple corn color resulted in determination C3G (Figure 4), pelargonidin-3-O-glucoside, and peonidin-3-O-glucoside (Figure 8) as the major compounds. Further biological results suggested that C3G and pelargonidin-3-O-glucoside are the active compounds against LNCaP cells [53].

##### Strawberry

Strawberry (*Fragaria* × *ananassa* Duch.) has been known as a fruit possessing diverse micronutrients (minerals, vitamin C, etc.), and non-nutrient elements (phenolic compounds). This plant predominantly contains ACD and ACN derivatives of malvidin, cyanidin, peonidin, delphinidin, pelargonidin, and petunidin [67]. Indeed, the renowned anti-inflammatory and antioxidant potentials of strawberry as major bioactivities can be attributed to the abovementioned phytochemicals [68].

**Figure 9:**
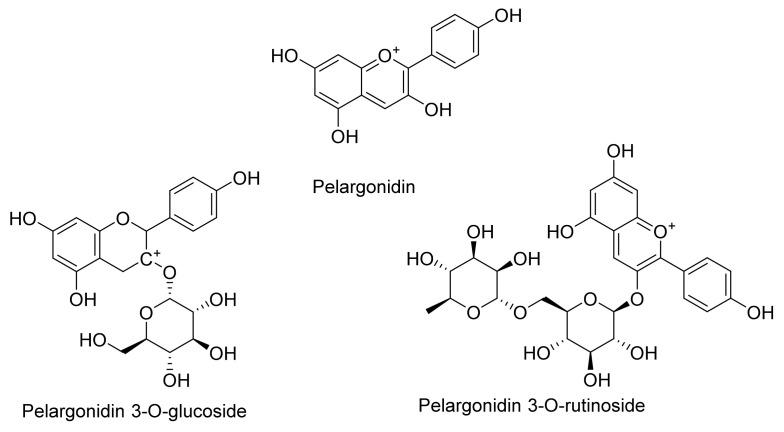
Chemical structure of major compounds presented in strawberry.

In a former investigation, phytochemical analysis of strawberry extracts identified C3G (Figure 4), pelargonidin, pelargonidin-3-O-glucoside, pelargonidin-3-O-rutinoside, aglycone and glycosylated flavonoids (Figure 9), as well as phenolic acids as the main compounds [39]. Quercetin, ellagic acid, 3,4,5-trihydroxyphenyl-acrylic acid, and C3G, as pure compounds at 100 µg/mL effectively inhibited the growth of the human prostate (LNCaP, and DU145) cancer cell lines, compared to the rest of pure compounds, while, anthocyanin and tannin-rich extracts of strawberry at 250 µg/mL demonstrated the best potency against these two cancer cells [39].

A polyphenol-rich extract plus anthocyanin or tannin-rich sub-fractions derived from a strawberry sample at 1.5 to 50 μg/mL were experimented against P21 (human prostate epithelial cell line), LNCaP and PC3 (tumor cell line). The strawberry extract (5 μg/mL) was cytotoxic on normal, and the tumor cell lines. The findings showed that the tannin-rich fraction was considerably more toxic to all tested cells than the ACN-rich fraction. As predicted, the prostate (LNCaP and PC3) tumor cell lines were more resistant to the extracts with around 50 μg/mL required for 50% survival reduction [40].

##### Cherry

The sweet cherry (*Prunus avium* L.) is characterized for its rich phytoconstituents particularly ACNs, hydroxybenzoic and hydroxycinnamic acids, carotenoids, and flavonoids [69]. In a previous study, the amounts of ACNs in the methanolic extracts of different sweet cherry samples were examined and the “late harvest” extract highly enriched in ACNs was selected for the proliferation, and apoptosis assessments on the neoplastic (LNCaP and PC3) and non-neoplastic (PNT1A) human prostate cells. HPLC-analysis confirmed that the most abundant phenolic compounds in the extract were the flavonoid quercetin-3-4-di-O-glucoside (24.61 ± 0.42 mg/100 g fresh fruit) and the anthocyanin C3G (Figure 4) (22.03 ± 0.74/100 g fresh fruit) [43].

The biological results exhibited that the cherry extract-treatment diminished the viability of neoplastic cells mostly at 2 µg/mL and non-neoplastic cells at 20 and 200 µg/mL, whereas it enhanced apoptosis in LNCaP. This was confirmed through caspase-9 increasing in LNCaP cells and decreasing in PC3 cells after the cherry extract treatment. In addition, such treatment did not significantly change Bax (proapoptotic, Bcl-2-associated X protein) protein expression in all cell lines. Anti-apoptotic protein Bcl-2 (B-cell lymphoma 2) was significantly decreased in PNT1A and LNCaP cells after exposuring to the cherry extracts with no remarkable alterations observed in PC3 cells. Besides, the glucose consumption was decreased in LNCaP cells (43%) and increased in PC3 (113%) when treated with the cherry extract. The PFK1 protein levels were significantly reduced in PNT1A- and PC3-treated cells, while LNCaP showed a significant increase in PFK1 protein expression. Moreover, the activity of lactate dehydrogenase (LDH) was improved to ~27% in the treated PNT1A cells while LDH activity was decreased in both neoplastic cells, LNCaP and PC3 to 49% and 60%, respectively [43].

##### *Ixora coccinea* 

In a previous study, the phytochemical screening showed that *Ixora coccinea* (jungle geranium) contains total phenolic, flavonoid, and anthocyanin contents with 128 mg, 20.63 mg, and 30 mg/100 g fresh weight, respectively. Although the analytical analysis demonstrated sinapic acid (21.96 mg) and myricetin (0.13 mg) as the major phenolics, the significant ACN content can not be ignored. According to the MTT evaluations, the fruit crude extract exhibited good anti-cancer potential against LNCaP.FGC cells with an IC_50_ value of 34.09 mg/mL after 24 h exposure [44].

##### Potato

Phytochemical studies showed that the *Solanum* spp. is rich in phenolic compounds including flavonoids such as kaempferol, naringenin, rutin, catechin, and epicatechin, however ACNs can be found in purple flesh varieties abundantly [70]. Some investigations have been designed in order to assess the potato extract’s capacities to affect PC cells.

In an experiment, the effects of the potato phenolic extract as well as organic acid, phenolic acid, and ACN-rich fractions against LNCaP and PC-3 were investigated. Potato extract and an ACN-rich fraction (at 5 mg chlorogenic acid eq/mL) were more potent compared to the rest of the extracts, where it inhibited cell proliferation, increased the cyclin-dependent kinase inhibitor p27, and induced apoptosis in both LNCaP and PC3 cells. Mitogen-activated protein kinase, c-Jun N-terminal kinase activation, as well as stimulated caspase-independent apoptosis through nuclear translocation of endonuclease G (Endo G) and apoptosis-inducing factor were observed [46].

In another study, it has been shown that the sweet potato extract has high content of polyphenols such as ACNs and phenolic acids. This extract significantly inhibited cellular proliferation of all tested prostate cancer cells named C4-2 (IC_50_ = 145 µg/mL), LNCaP (IC_50_ = 150 µg/mL), DU145 (IC_50_ = 280 µg/mL), and PC3 (IC_50_ = 315 µg/mL), while demonstrated no anti-proliferative effect against normal prostate epithelial cells PrEC and RWPE-1 with IC_50_ of 1000 and 1250 µg/mL, respectively [52].

##### *Acanthopanax senticosus*

**Figure 10:**
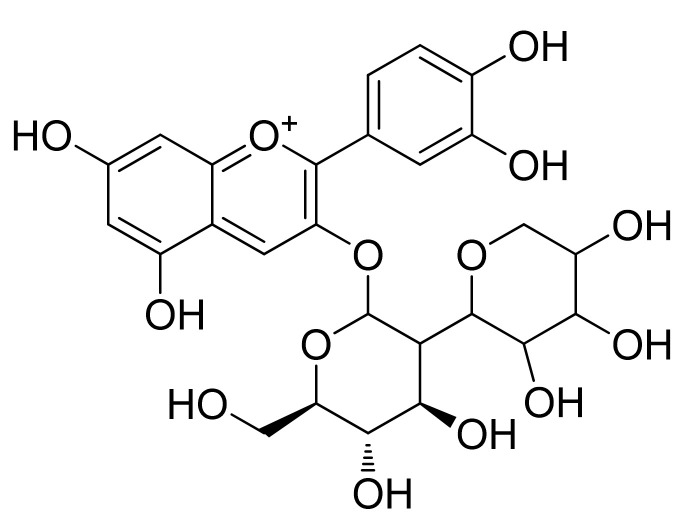
Chemical structure of cyanidin-3-O-(2″0-O-xylosyl)-glucoside.

In an acidified methanolic extract of *Acanthopanax senticosus* (Siberian Ginseng), cyanidin-3-O-(2″-O-xylosyl)-glucoside (Figure 10) was identified, representing 5.2 mg/g of extract. This ACN exerted highest cytotoxic effect against LNCap cell (IC_50_ of 5.2 µg/mL), in comparison with the other studied cell lines MOLT-4F (leukemia), and ACHN (renal) with IC_50_ 11.2 and 22.5 µg/mL, respectively [54], where further studies can promisisngly indicate the mechanism of actions.

##### *Lycium ruthenicum*

**Figure 11:**
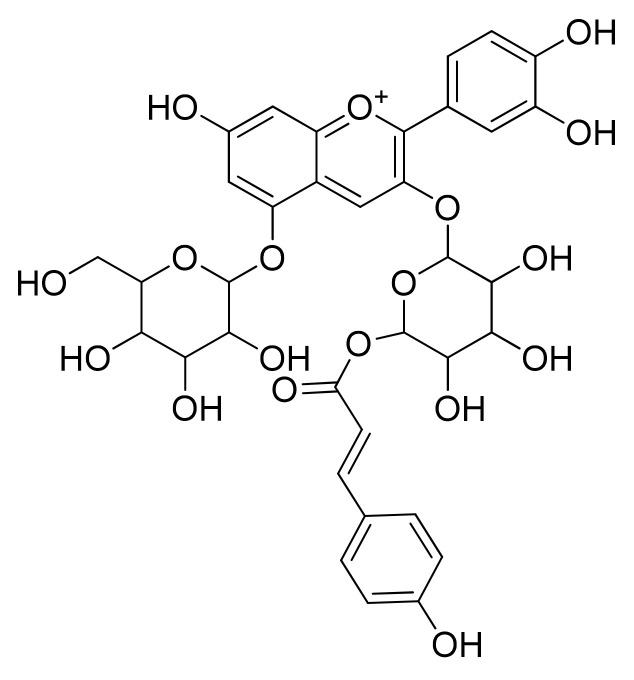
The extracted anthocyanin monomer from *Lycium ruthenicum* Murray namely petunidin 3-O-[6-O-(4-O-(trans-p-coumaroyl)-α-l-rhamnopyranosyl)-β-d-glucopyranoside]-5-O-[β-d-glucopyranoside].

The fruit of *Lycium ruthenicum* commonly is famed as black wolfberry. ACNs have been reported as the main chemical ingredient, apart from phenolic acids, flavonoids, and alkaloids [71]. The extracted monomer ACN (Figure 11) from *Lycium ruthenicum* Murray (PGG: petunidin 3-O-[6-O-(4-O-(trans-*p*-coumaroyl)-α-l-rhamnopyranosyl)- β-d-glucopyranoside]-5-O-[β-d-glucopyranoside]) inhibited cell proliferation, induced apoptosis, and promoted cell cycle arrest in the S phase dose-dependently at 100, 200, and 400 µg/mL. Further analysis indicated that this monomer significantly increased the expression of phosphatase and tensin homolog deleted on the chromosome (PTEN) to induce apoptosis, stimulate the overproduction of ROS, and activate the PI3K/Akt mediated caspase 3 pathway on prostate cancer DU-145 cells [55].

##### *Hibiscus sabdariffa*

It has previously been reported that various biological properties of *Hibiscus sabdariffa* is correlated to its phenolic compounds, majorly ACNs (delphinidin-3-sambubioside and cyanidin-3-sambubioside), as well as some phenolic acids (protocatechuic acid), and other natural acids (e.g., hibiscus acid and hydroxycitric acid) [72].

Anticancer potencies of the *H. sabdariffa* Linne leaf, as a waste product, has been investigated. The extract exhibited high content of polyphenols, including catechin (4.25%) and ellagic acid (28.20%) with 2% total ACN. The leaf extract at 0.5, 1.0 and 2.5 mg/mL induced anticancer potential against PC3 and DU145 especially LNCaP. Furthermore, the extract and ellagic acid demonstrated IC_50_ values of 2.5 mg/mL and 100 µM against LNCaP cells, respectively. Molecular assessment of LNCaP cells after treatment with the *H. sabdariffa* L. leaf extract proposed the two death pathways including intrinsic (Bax/cytochrome c-mediated caspase 9) and extrinsic (Fas-mediated caspase 8/t-Bid) apoptotic pathways [49].

##### Blueberry

Elaboration of anti-tumor activities of Blueberry (*Vaccinium* L.), as one of the main sources of ACDs and ACNs, has been targeted by many researchers. The mechanism to downregulate MMP activity in DU145 human PC during treatment with the crude extract, anthocyanin-enriched and proanthocyanidin-enriched fractions from lowbush blueberry were examined at 0.1, 0.5, and 1.0 mg/mL. The downregulation of MMPs activity and up-regulation of tissue inhibitors of metalloproteinases (TIMP) activity in DU145 cells were evinced in three tested extracts; however, proanthocyanidin-enriched were more potent compared to the other extracts tested. Multiple mechanisms in down-regulating MMPs involve an increase in TIMP-1 and TIMP-2 activities via inhibition of protein kinase C (PKC) and mitogen-activated protein (MAP) kinase pathways without affecting PI-3 kinase [50].

In another study, phytochemical profiles of araca (yellow and red), and pitanga (red and purple), as well as strawberry, blackberry, and blueberry were analysed. Among the tested extracts, blueberry showed the highest total ACN content with 1202 mg/C3G kg fresh fruit, while strawberry exhibited the highest total phenolic content (13.55 g equivalent of gallic acid/kg fresh fruit). Strong positive correlations were detected between the total ACN content of the tested extract and antioxidant potential but not with total phenolic content. Screening of the extracts at maximum concentration of 0.5 mg/mL showed no cytotoxicity against DU145 cancer cell line [35].

The *V. myrtillus* extract and recombined standard mixture of its main native polyphenols have shown beneficial effects against hormone-dependent and hormone-independent PC cell lines. The extract at 1/800 (*v*/*v*) corresponding to 14.15 C3G equivalents/mL of culture medium decreased the proliferation of anchorage-dependent prostate cancer cell lines in a dose-dependent manner. Moreover, the *V. myrtillus* extract at 1/100 (*v*/*v*) demonstrated a significant growth inhibitory effect towards PrEC and DU145 cells with high apoptotic rates compared to control. The recombined standard mixture of its main native polyphenols also showed the same activity as the extract with induction of growth-inhibitory effect under hypoxia [36].

##### Blackberry and Raspberry

Blackberry (*Rubus fruticosus*) belonging to Rosaceae family is enriched in phenolic compounds mostly flavonoids including flavonols, ACNs, and ellagitannins [73]. It has previously been reported that this fruit due to the presence of these phenolics, is a potent natural agent for the treatment of the age-related neurodegenerative diseases, apart from its high antioxidant effect [74].

Zambrano et al. focused on cytotoxic properties of blackberry and soursop and their formulated beverage named F2 (yogurt + soursop and blackberry pulps + sweeteners), and F3 (yogurt + soursop and blackberry pulps + sweeteners + pH regulator). According to MTT assessments, F2 and F3 formulations showed 44.38 ± 0.29% and 56.05 ± 0.68% cell viability at 5% *v*/*v* concentration against PC3. The cell viability reduction with soursop and blackberry pulps, with values of 9.64 ± 0.69% and 11.70 ± 0.29% at the same concentration against PC3 were more significant [42].

Ellagic acid and ACN constituents including urolithin A and protocatechuic acid of black raspberry as well as the black raspberry (*Rubus occidentalis*) extract have been evaluated for the interference with chemotherapy docetaxel and cabazitaxel for castrate-resistant prostate cancer (CRPC). None of the extracts altered the enzyme responsible of CYP3A4 for catabolizing taxanes. Also, protocatechuic acid and the black raspberry extract were not able to enhance the effects of taxane chemotherapeutics, taxane metabolism, or mechanisms of resistance; however, the microtubule polymerizing effect of ellagic acid in vitro might interfere with microtubule polymerization induced by taxane drugs which reduce their effectiveness, although dietary ellagic acid did not alter the tumor growth inhibition by docetaxel of xenografted 22Rv1 cells. It seems that consumption of black raspberry during taxane chemotherapy could not provide a therapeutic benefit, it is safe and not likely to reduce drug effectiveness or increase toxicity while potential adverse interaction between taxane chemotherapy and ellagic acid was detected [75].

##### Pomegranate

As one of the oldest edible fruits, pomegranate (*Punica granatum* L., Punicaceae family) indicated high antimicrobial, antioxidant, and anti-carcinogenic potencies. Many studies evaluated the phytochemicals of different variety, reporting that besides tannins, ellagic acids, and flavonoids, this valuable fruit is a rich source of ACDs/ACNs; from them the ACN analogs of cyanidin (cyanidin 3,5-O-diglucoside and C3G), pelargonidin (pelargonidin 3,5-O-diglucoside and pelargonidin 3-O-glucoside), and delphinidin (delphinidin 3,5-O-diglucoside and delphinidin 3-glucoside) can be highlighted [76,77].

Previously, it has been reported that constitutive NF-κB activation is observed in androgen-independent PC. In this context, the pomegranate extract effect was evaluated towards the progression of PC. The pomegranate extract mediated NF-κB blockade and decreased the cell viability of PC cell lines in a dose-dependent fashion in vitro. Importantly, the increase in NF-κB activity during the transition from androgen dependence to androgen independence in the xenograft model was overturned by the extract [45].

##### Black Carrots

Studies showed that the dark color of black carrot (*Daucus carota* ssp. *sativus* var. *atrorubens* Alef.) is due to the ACNs richness [78]. In a previously performed experiment, the PC3 cancerous cell lines have been treated with the extracts of black carrot specimens in 6.25, 12.5, 25, 50, and 100 μg/mL doses of the extracts. MTT assessments recorded moderate cell viabilities with the values of 58 and 77% at a concentration of 100 μg/mL [48].

#### 4.2.2. In Vitro Screening of Pure Anthocyanidin/Anthocyanin Derivatives on Prostate Cancer Cell Lines

In few studies potential effects of delphinidin and cyanidin-3-O-β-glucopyranoside (C3G) on prostate malignancy have been analyzed; however due to the remarkable activities of the extracts as above overviewed, further studies can complement those effects.

##### Delphinidin

**Figure 12:**
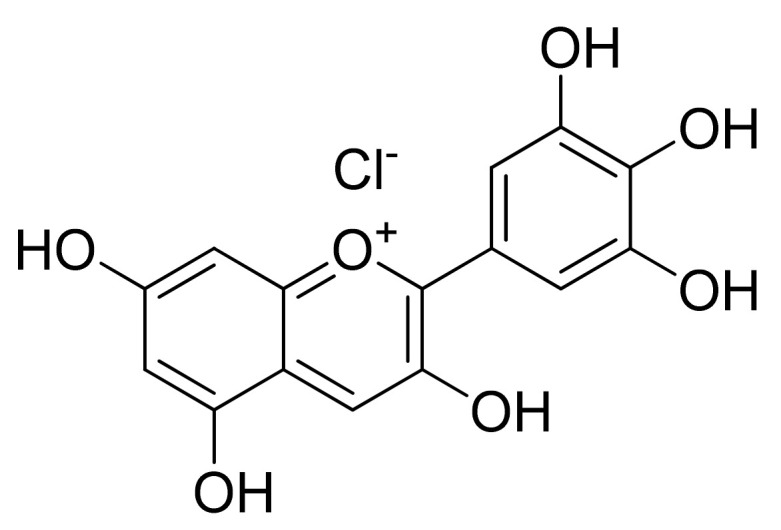
Chemical structure of delphinidin.

Delphinidin (Figure 12, 3,3′,4′,5,5′,7-hexahydroxyflavylium), an ACD compound, which can be found abundantly in berries and red wine demonstrated a variety of bioactivities, dominantly antioxidant, anti-inflammatory, and anti-tumor properties [79]. In line with anticancer assessments of this compound, some researchers have also evaluated its capacity to suppress prostatic cancer cells.

In a previous study, the apoptotic properties of delphinidin against human prostate cancer cells LNCaP, C4-2, 22RN1, and PC3 were analyzed, without bearing any substantial effect on normal human prostate epithelial cells. Delphinidin decreased the phosphorylation of IKB kinase (NEMO), phosphorylation of nuclear factor-KB (NF-κB) inhibitory protein IKBA, phosphorylation of NF-κB/p65 at Ser536 and NF-κB/p50 at Ser529, plus reducing NF-κB/p65 nuclear translocation and NF-κB DNA binding activity in vitro [58].

In another study conducted by Malik et al., the treatment of delphinidin (30–300 μM) on PC3 cells for 48 h resulted in a dose-dependent inhibition of cell growth and/or cell viability through induction of cyclin kinase inhibitors p21/WAF1 and p27/KIP1. Also, the downregulation of cyclin E, D1, and D2 and cyclin-dependent kinase 2, 4, and 6 were recorded. Apoptosis induction during treatment with delphinidin was validated via decreasing level of anti-apoptotic protein Bcl-2 and increasing the pro-apoptotic protein Bax. Supportively, significant activation of caspases and procaspase-3, -6, -8, and -9 as well as the release of cytochrome c from the mitochondria to the cytosol were measured. Finally, flow cytometry and morphological changes confirmed the apoptosis induction via treatment with delphinidin [80].

##### Cyanidin-3-O-Glucoside

Cyanidin-3-O-glucoside (syn. cyanidin-3-O-β-glucopyranoside: C3G) (Figure 4) as a natural antioxidant ACN indicated anti-proliferative effects at 3–200 µM through activation of caspase-3 and induction of p21 protein expression. After the C3G treatment, the clear increase of DNA fragmentation in the DU145 and LnCap human prostatic cancer cell lines with no cytotoxic effect against normal human epithelial cells were observed. Furthermore, an increase in the levels of tumor suppressor P75NGFR revealed a possible role of this pure compound in the acquisition of a normal-like cell phenotype [57].

### 4.3. In Vivo Experiments Evaluating the Effectiveness of Anthocyanidins/Anthocyanins on Prostate Cancer

Preclinical in vivo experimental data was gained from rodent models in mice including athymic nude mice implanted with PC3 and stress provoked benign prostate hyperplasia (BPH); however, animal models in rats include BPH induced by testosterone derivatives such as testosterone enanthate or testosterone propionate. Followings describe the animal studies evaluating the impacts of ACDs/ACNs and natural sources of these compounds on PC cells (Table 2).

#### 4.3.1. Mice Model Experiments

Diet with delphinidin suppressed the tumor growth in the athymic nude mice implanted with PC3 cells. In addition, it controlled the protein levels of Bax and Bcl-2, and inhibited the protein levels of cyclin D1 and NF-κB. Furthermore, it suppressed PCNA and Ki67 expression (markers of proliferation) in PC3 originated tumors in athymic nude mice; suggesting the anti-proliferative efficacy of delphinidin [81]. Supportively, the delphinidin administration (2 mg, i.p. thrice weekly) to athymic nude mice implanted with PC3 cells showed a significant decrease in the expression of NF-κB/p65, Bcl-2, Ki67, and PCNA [58].

Oral administration of sweet potato greens extract (SPGE) significantly inhibited growth and progression of prostate tumor xenografts by 69% in athymic nude mice, as shown by non-invasive real-time bioluminescent imaging and tumor volume measurements. The SPGE treatment reduced the cyclin D, E and D1 expression, and rose the p21 expression in tumor cells. Also, the caspase-3/7 activity and cleaved caspase-3 expression was higher in SPGE-treated tumors in comparison with control, while it proposes the in vivo anti-proliferative response of the SPGE. Moreover, this extract showed no toxicity to quickly divide normal tissues such as bone marrow and gut [52]. Sweet potato extract also inactivated Bcl-2 as well as upregulated BAX, cytochrome c release, and triggered downstream apoptotic signaling. Oral administration of 400 mg/kg extract inhibited growth and progression of prostate tumor xenografts by ~70% in nude mice [52].

A supplementary diet with the rich ACNs extract (AE) of bilberry (*Vaccinium myrtillus* L.), administered to stress-provoked BPH in mice, had the protective effects against stress-induced oxidative injury by reducing lipid peroxidation content, elevating glutathione content, oxygen radical absorbance capacity, as well as glutathione peroxidase and superoxide dismutase activities. These results showed the antioxidative activity of AE involved in the amelioration of BPH [84].

Wang et al. investigated the antitumor effect of anthocyanin C3G extracted from Chinese bayberry fruit (*Myrica rubra* Sieb. et Zucc.), on a nude mouse tumor xenograft model. C3G inhibited the growth of SGC-7901 tumor xenografts. The average tumor volume on the 18th day were measured 771.5 mm^3^, 211.7 mm^3^, 384.1 mm^3^ and 276.0 mm^3^, in the control group, tegafur-treated group (positive control), C3G treatment groups with low dose and high dose, respectively. Moreover, C3G revealed ability to inhibit the cell cycle of SGC-7901 tumor xenografts in the C3G treatment groups by obvious positive p21 staining in cell nuclei in a dose-dependent manner. The mRNA expression and protein expressions of KLF6 and p21 were significantly augmented in the C3G treatment group in comparison with normal group, representing the vital role of KLF6 in the tumor xenograft inhibition in vivo. However, the p53 protein did not alter, but Cyclin D1 and CDK4 mRNA and proteins levels were reduced [91].

Ha et al. evaluated the apoptosis and tumor growth of ACNs extracted from the black soybean on DU-145 tumor xenografts established in athymic nude mice. Black soybean (*Glycine max* L. Merr) has been known for its phenolic rich content mainly flavonoids (i.e., ACDs), stilbenes, lignans, and phenolic acids. It has demonstrated a variety of biological potencies including anti-tumor, anti-diabetic, and for the treatment of cerebrovascular, neurodegenerative, and cardiovascular diseases [92]. It has been exhibited that 2 weeks oral treatment with daily 8 mg/kg of ACN increased apoptosis significantly in a dose-dependent manner. Also, the decrease in p53 and Bcl-2 expressions with the increased Bax expression, as well as a lessening in PSA and AR co-expression was observed [87].

In another study, Lamas et al. evaluated the impact of two doses of the patented jaboticaba peel extract (PJE) on inflammation and oxidative stress in the prostate of high-fat-fed (HFD) aging or aging mice. The PJE decreased the contents of oxidative-stress parameters (4-hydroxynonenal, glutathione peroxidase 3, glutathione reductase, and catalase), inflammatory mediators phosphorylated signal transducers and activators of transcription 3 (pSTAT-3), tumor necrosis factor α (TNF-α), cyclooxygenase 2 and CD3^+^ T cells’ number, which were correlated to preservation of the glandular morphological integrity of prostate in HFD-fed aging and aging mice. Nevertheless, merely the high PJE dose ameliorated the toll-like receptor 4 (TLR4), and nuclear factor κB (NF-κB) contents in aging mice, and IL-1β, SOD2, and IL-6 levels in HFD-aging mice. Authors stressed that PJE had a dose-dependent impact on regulating oxidative-stress and inflammation in HFD-fed aging and aging mice prostate [90].

#### 4.3.2. Rat Model Experiments

Oral administration of the ACNs extracted from black soybeans significantly reduced prostate weight in a rat model of BPH induced by testosterone propionate (TP). The mean prostate weight for the control, BPH-induced rats, and rats receiving 40, 80, and 160 mg/kg ACNs were 674.17 ± 28.24 mg, 1098.33 ± 131.31 mg, 323.00 ± 22.41 mg, 324.00 ± 26.80 mg, and 617.50 ± 31.08 mg, respectively. The average prostate weight in the ACNs-treated groups were markedly lower rather than in the BPH-induced rat, while the apoptotic body counts were significantly higher than in the BPH-induced animal. These findings propose that ACNs may be useful in reducing the volume and inhibiting the proliferation of the prostate [82].

In another study, Jang et al. showed that oral administration of an ACN extracted from the seed coat of the black soybean prevented apoptosis in the prostate cells in andropause animal model by bilateral orchiectomy. Despite reporting no remarkable changes in prostate weight between andropause and andropause plus anthocyanin-treated groups, the apoptotic index was meaningfully lesser in andropause plus anthocyanin-treated groups compared to the andropause group. The oxidative stress in the ACN-administered group was reduced rather than in the andropause group [83].

A diet with ACNs extracted from black soybean may be useful in treating chronic bacterial prostatitis (CBP) rat model. The ACNs plus ciprofloxacin group indicated a significant reduction in improvement of the prostatic inflammation and bacterial growth in comparison with the ciprofloxacin group; suggesting that ACNs may have antimicrobial and anti-inflammatory effects [85].

Seoritae is a black soybean that contains high isoflavone and anthocyanin compounds. Oral administration of seoritae extract (SE) previously demonstrated beneficial effects on the loss of prostate weight and prostate proliferation in the BPH induced by testosterone enanthate in a rat model. The SE treatment groups indicated a significant reduction in prostate weight, apoptosis (caspase-3), oxidative stress markers (8-hydroxy-2-deoxyguanosine and SOD), and 5-α reductase activity compared to the non-treated BPH group [86].

In another research, oral administration of polymerized anthocyanin (PA) from grape skin was able to reduce the prostate weight in the TP-induced BPH rats. PA ameliorated serum dihydrotestosterone (DHT) levels, and the 5-α reductase type 2, androgen receptor, steroid receptor coactivator 1, proliferating cell nuclear antigen (PCNA), prostate-specific antigen, and cyclin D1 expression in prostate tissues. Furthermore, PA downregulated and upregulated the expressions of Bcl-2 and Bax, respectively, in the prostate tissues of rats with BPH. Authors stated that PA may be a possible natural agent for BPH treatment [88].

A diet with the extract of *Aronia melanocarpa* in TP-induced BPH Wistar rats attenuated prostate enlargement and decreased the levels of 5 α-reductase and DHT in prostate tissue and serum. The PCNA expression in prostate tissue was significantly suppressed in the *A. melanocarpa*-treated group. These results displayed that the extract (including ACNs) weakened the progression of testosterone-induced prostatic hyperplasia, and recommended that *A. melanocarpa* has beneficial potential to treat prostate enlargement and BPH [89].

A diet with 1% hexane insoluble fraction from a purple rice ethanolic extract (PRE-HIF) indicated a reduction in the prevalence of adenocarcinoma in the lateral lobes of the prostate and markedly higher percentage of low-grade prostatic intraepithelial neoplasia in transgenic rat for adenocarcinoma of prostate model. In addition, 1% PRE-HIF diet meaningfully repressed the tumor growth in a rat CRPC xenograft model of the PCai1 cells. Additionally, cyclin D1, androgen receptor, fatty acid synthase, and cdk4 expression were reduced while AMP-activated protein kinase α activation, and p38 mitogen-activated protein kinase upregulated in the prostate tissues of PRE-HIF-treated rats and CRPC tumors. Authors stated that the PRE-HIF can inhibit the progression of CRPC and PC by blocking the cell proliferation and metabolic pathways [60].

### 4.4. The Efficacy Assessment of Anthocyanidins/Anthocyanins on Prostate Cancer through Clinical Trials

In three clinical trials, the efficacy of ACDs or ACDs rich fruits have assessed, while 198 prostatic cancer men were targeted in those studies (Table 3). In a clinical trial, the effect of black raspberry (*Rubus occidentalis*) in different concentrations were evaluated on 56 men newly diagnosed with resectable prostate cancer. According to the results, during 8 weeks of black raspberry storage, no significant changes in bioactivities were observed, whereas with both dosages of 10 and 20 g black raspberry/day in nectar and confection products were accepted to be considered for further prostate cancer clinical trials [93].

Inflammation of the bladder which is called acute radiation cystitis, is defined as a prevalent side effect of external beam radiation therapy, used for the treatment of prostate cancer [94]. In two double-blinded randomized placebo-controlled trials, the impact of ACNs on cystitis of the patients during radiation therapy treatment have been investigated.

Hamilton et al. selected 41 patients with normal mainly New Zealand European diet, who were receiving image guided intensity modulated radiation therapy for the treatment of prostate cancer. The patients were taken one capsule of cranberry (*Vaccinium macrocarpon*) per day after breakfast for two weeks post radiation therapy treatment. Consequently, 65% and 30% of patients taking the capsules developed cystitis and severe cystitis, thus taking of cranberry is suggested for patients receiving radiation therapy, specifically with low hydration regimens or baseline urinary symptoms [94].

In another clinical trial, 101 men were chosen, who were receiving beam radiation therapy for the treatment of their prostate or prostate bed. After taking two capsules containing cranberry (*Vaccinium macrocarpon*) daily, during radiation therapy, no remarkable effect on RICAS (novel radiation induced cystitis assessment scale) was observed in comparison with the taking capsules and placebo groups. Therefore, it is suggested that analysis of RICAS can be implemented for measuring severe cystitis in prostate cancer therapy [95]. Since the fruits analysed in the abovementioned studies are well-known as rich sources of ACDs/ACNs, thus the effects can accordingly be related to those compounds, however further studies might be interesting to assess their effects in pure forms.

## 5. Conclusions and Perspectives

Several successful anti-tumor drugs have been derived and developed from natural sources, more effective than synthetic counterparts. So far, paclitaxel and the analogs docetaxel and cabazitaxel, vincristine, vinblastine and their derivatives vindesine and vinorelbine, camptothecin and analogs belotecan, topotecan, and irinotecan, besides podophyllotoxin and analogs etoposide and teniposide are the well-known phytochemical drugs for the remedy of diverse cancer types. In addition, the other natural sources have further proven be worth to investigate their secondary metabolites; notably doxorubicin, epirubicin, daunorubicin, bleomycin, and dactinomycin have been isolated and developed from bacteria, prescribed as potent natural antitumor medications [96,97]; therefore discovering potent antitumor natural products is still a promising approach for researchers in diverse fields.

Through a comprehensive literature overview, the present study described the beneficial effects of ACDs/ACNs, and the plants rich in these compounds on the treatment and prevention of prostate cancer. The preclinical including in vitro and animal studies confirmed the valuable effects of these secondary metabolites. Overall, the ACNs indicated higher anti-prostate cancer activities compared to the aglycone derivatives (ACDs) and plant crude extracts, whilst among them cyanidin-3-O-glucoside is the most studied compound possessing highest potency, which can abundantly be found in rice, maize, cherry, berries, etc.

ACDs/ACNs exhibited reducing effects on survival, Snail and pSTAT3 expression, inhibiting MMP activity, as well as potencies in enhancing p21 expression, apoptosis induction, and activating caspase-3, which were experimented on various prostatic cancer cells (PC3, PCai1, CRPC, LNCaP, DU145 human cell lines).

Through animal studies, these compounds and their rich content extracts have further demonstrated potent effects on suppressing prostatic malignancy. The major pathways including anti-inflammatory (via reducing NF-κB/p65 and PCNA expression), and apoptosis induction (via reducing Bcl-2 and increasing Bax expression), as well as physical effects on tumor cells i.e., attenuating the tumor weight and prostate hyperplasia can be highlighted as effective traits of these natural products.

Fruits are inseparable part of the human diet, while wide range of them are as sources of ACDs/ACNs. Through preclinical and clinical studies, the preventive effects of these natural class of compounds against various cancer types have been confirmed. Lin et al. overviewed their high cancer chemopreventive potencies in accordance with the remarkable related biological effects (e.g., anti-inflammation, antioxidant, anti-proliferation, anti-invasion, inducing cell cycle arrest, apoptosis, autophagy, anti-metastasis, etc.) [9]. As a consequence of the present review, aside from potent activities of ACDs/ACNs for the treatment of PC, their abilities to prevent this malignancy are also acknowledged.

Since most of the studies have evaluated the crude extracts, further in vitro and in vivo experiments can potentially discover promising bioactive derivatives of these natural compounds, as adjuvant or merely consumption for the prevention and treatment of prostate cancer, while they might be of interest by phyto-pharmaceutical and food industries. Moreover, the efficacy assesments through clinical trials as well as toxicological studies of them in pure forms can confirm their potential effects on prevention and treatment of prostate cancer.

## Figures and Tables

**Table 1:** In vitro anti-tumor assessments of anthocyanidins/anthocyanins in pure forms and in plant rich extracts towards prostate cancer cells.

Anthocyanin Source	Cell Line	Effect	Ref
Purple rice extract	LNCaP	Extract at 200 µg/mL significantly reduced the viability of prostate cancer cells after 48 h	[33]
Muscadine grape skin extract	LNCaP	The extract decreased Snail and pSTAT3 expression and abrogated Snail-mediated CatL activity, migration, and invasion	[34]
	ARCaP-E		
Brazilian native fruits, pitanga (red and purple) and araçá (yellow and red), as well as strawberry cultivars Albion, Aromas, and Camarosa, blackberry cultivar Tupy, and blueberry cultivar Bluegen	DU145	No cytotoxicity was found	[35]
*Vaccinium myrtillus* berry extract	LNCaP	Apoptotic rate (early and late) was statistically higher in tested cell lines exposed to *Vaccinium myrtillus* berry extract compared to control.	[36]
	PC3 and DU-145	Anchorage-dependent and anchorage-independent growth inhibition were seen	
American cranberry (*Vaccinium macrocarpon*) extract	DU145	10, 25 and 50 µg/mL of the extract significantly decreased the cellular viability of DU145 cells.	[37]
		The extract at 25 and 50 µg/mL also lessened the proportion of cells in the G2-M phase of the cell cycle and increased the proportion of cells in the G1 phase after 6h in prostate carcinoma.	
Red cabbage	DU145	After 48 and 72 h 1, 2, 3, 4 and 5% juice reduced the proliferation of prostate cancer cell lines compared to the control group.	[38]
	LNCaP		
Strawberries (*Fragaria* × *ananassa* Duch.)	LNCaP	Crude extracts (250 µg/mL) and pure compounds including C3G, pelargonidin, pelargonidin-3-glucoside, and pelargonidin-3-rutinoside (100 µg/mL) inhibited the growth of tested cancer cells significantly	[39]
	DU145		
Strawberry	P21	The strawberry extract was cytotoxic with doses of ~5 μg/mL causing a 50% reduction in cell survival in both the normal and the tumor lines.	[40]
	P21 tumor cell line 1 and 2		
	LNCaP		
	PC3		
Blue Maize (*Zea mays* L.)	PC3	PC3 treated with 5 mg/mL of acidified and non-acidified extracts demonstrated cell viability in the range of ~30 to 70%	[41]
Blackberry (*Rubus glaucus* B.) and soursop (*Annona muricata* L.)	PC3	Blackberry pulp and soursop pulp recorded cytotoxic effect with an IC_50_ of 1.81 ± 1.68% *v*/*v* and 1.34 ± 1.06% *v*/*v*, respectively	[42]
Sweet Cherry	PNT1A	The extract reduced the cell viability after 72 h at 2, 20, and 200 µg/mL compared to the control group MTT assay.	[43]
	LNCaP		
	PC3		
*Ixora coccinea* (fruits)	LNCaP.FGC	The fruit extract exhibited anticancer activity against LNCaP.FGC cells with an IC_50_ value of 34.09 mg/mL.	[44]
Pomegranate extract	LNCaP	240 mL solution inhibited NF-κB and cell viability of prostate cancer cell lines in a dose-dependent fashion.	[45]
	LAPC4		
	DU145		
Potato phenolics and their fractions	LNCaP	5 mg chlorogenic acid eq/mL inhibited cell proliferation and increased the cyclin-dependent kinase inhibitor p27 levels in both LNCaP and PC3 cells.	[46]
	PC3		
Thai rice (*Luempua cultivar*)	PC3	Thai rice showed cell viability with IC_50_ value of 167.8 ± 0.06 µM.	[47]
Black carrots	PC3	Cell viabilities were between 58 and 77% at a concentration of 100 μg/mL of extract.	[48]
*Hibiscus sabdariffa*	LNCaP	IC_50_ = 2.5 mg/mL against LNCaP cells viability.	[49]
	PC3		
	DU145		
Lowbush blueberry	DU145	Inhibition of MMP9 activity at 0.5 and 1.0 mg of crude fraction/mL was seen. Also, 1.0 mg/mL extract decreased the gelatinolytic activity of the activated isoforms of MMP-2 and complete inhibition of the pro-MMP-2, with an increase in TIMP-1 and TIMP-2 action.	[50]
(*Vaccinium angustifolium*):			
Jaboticaba peel	PC3	The non-polar extract was the most active agents against prostate cancer cells with GI_50_ = 13.8 μg/mL.	[51]
Sweet potato (*Ipomoea batatas*)	C4-2	The extract significantly inhibited cell proliferation of all prostate cancer cells with IC_50_ values in the range of 145–315 µg/mL. IC_50_ of extract in normal prostate epithelial cells (PrEC and RWPE-1) was between 1000 and 1250 µg/mL.	[52]
	LNCaP		
	DU145		
	C4-2B		
	PC3		
	PrEC		
	RWPE-1		
Purple corn	LNCaP	Purple corn color at 50 and 100 ppm inhibited the proliferation of LNCaP cells by decreasing the expression of cyclin D1 and inhibiting the G1 stage of the cell cycle.	[53]
*Acanthopanax senticosus* (Siberian Ginseng)	LNCap	Cyanidin-3-O-(2″-O-xylosyl) glucoside showed potent anticancer effects with IC_50_ of 5.2 µg/mL	[54]
petunidin 3-O-[6-O-(4-O-(trans-p-coumaroyl)- α-L-rhamnopyranosyl)- β-D-glucopyranoside]-5-O-[ β-D-glucopyranoside] extracted from *Lycium ruthenicum* Murray	DU145	The IC_50_ viability of the compound against DU145 cells was about 361.58 µg/mL. The main anthocyanin monomer also inhibited cell proliferation, induced apoptosis, and promoted cell cycle arrest at the S phase.	[55]
Cranberries (*Vaccinium macrocarpon* Ait.)	RWPE-1, RWPE-2, 22Rv1	Total cranberry extract and all fractions (200 µg/mL) showed ≥50% antiproliferative activity against prostate cancer cells. Total polyphenols fraction as the most active one exhibited RWPE-1, 95%; RWPE-2, 95%; 22Rv1, 99.6% anti-proliferation potentials.	[56]
C3G	DU145	Compound produced significant anti-proliferative effects at 6 µM compared to the control group. Also, activation of caspase-3 and induction of p21 protein expression were seen at 50 and 100 µM.	[57]
	LnCap		
Delphinidin	PC3	Pure compound at 30–300 μM resulted in the induction of cyclin kinase inhibitors p21/WAF1 and p27/KIP1, down-regulation of cyclin E, D1, and D2, and cyclin-dependent kinase 2, 4, and 6.	[58]

ROS: reactive oxygen specie; C3G: cyanidin 3-O-glucoside.

**Table 2:** Animal studies evaluating the effects of pure anthocyanidins/anthocyanins and extracts rich in these compounds on prostate cancer.

Applied Species	Diet	Supplement	Anthocyanin Dosage	Effect/Observation	References
Athymic (nu/nu) male nude mice	An autoclaved diet ad libitum	Delphinidin	2 mg/animal in 100 AL of 1:10 ratio of DMSO three times a week for 12 weeks	Reduced the expression of NF-κB/p65, Bcl-2, Ki67, and PCNA	[81]
12-week-old Sprague-Dawley male rats	A diet ad libitum	Anthocyanin extracted from black soybean	40, 80, and 160 mg/kg of anthocyanin daily for 4 weeks	Decreased the volume and suppressing the proliferation of the prostate	[82]
6-week-old male nude mice	Normal diet	Polyphenol-rich sweet potato greens extract	400 mg/kg polyphenol-rich sweet potato greens extract daily for 6 weeks	Inhibited growth and progression of prostate tumor xenografts by 69% in nude mice	[52]
12-week-old male Sprague-Dawley rats	n.d	Anthocyanin extracted from the seed coat of the black soybean	160 mg/kg of anthocyanin daily for 8 weeks	Prevented the rapid prostatic cell death by apoptosis in the prostate in an animal model of andropause	[83]
7-week-old male Kunming mice	Standard diet	Anthocyanin extract from bilberry	200 mg/kg of anthocyanins extract from bilberry (*Vaccinium myrtillus* L.)	Enhanced the therapeutic effect of Pollen of *Brassica napus* L. on stress-provoked benign prostatic hyperplasia	[84]
Male Sprague-Dawley rats		Anthocyanin extracted from black soybean	50 mg/kg of anthocyanin extracted from black soybean twice a day for 2 weeks	Showed the anti-inflammatory and antimicrobial effects, as well as the synergistic effect with ciprofloxacin in chronic bacterial prostatitis	[85]
16-week-old Sprague Dawley male rats		Seoritae extract including isoflavone and anthocyanin	228 and 457 mg/kg of seoritae extract in 1 mL distilled water daily for 5 weeks	Reduced the prostate weight, oxidative stress, apoptosis, and 5α-reductase activity	[86]
6-week-old male BALB/c nude mice		Anthocyanin from black soybean	8 mg/kg of anthocyanin dissolved in 1 mL of distilled water daily for 14 weeks	Inhibited the progression of prostate cancer in a xenograft model.	[87]
8-week-old male Sprague–Dawley rats	Standard laboratory diet	Polymerized anthocyanin from grape skin	100 mg/kg of polymerized anthocyanin from polymerized anthocyanin daily for 4 weeks	Reduced the prostate weight in rats with testosterone propionate–induced BPH, decreased the AR, 5AR2, SRC1, PSA, PCNA, and cyclin D1 expression in prostate tissues, ameliorated the BPH-mediated increase of Bcl-2 expression, and increased the Bax expression.	[88]
7-week-old male Wistar rats	A diet ad libitum	*Aronia melanocarpa* containing C3G and cyanidin-3-xylose	100 mg/kg of *Aronia melanocarpa* extract daily for 6 weeks	Attenuated the development of testosterone-induced prostatic hyperplasia	[89]
Male FVB mice	Standard diet	Brazilian berry extract (*Myrciaria jaboticaba*)	2.9 and 5.8 g/kg of jaboticaba peel extract daily for 60 days	Exerted a dose-dependent effect controlling inflammation and oxidative-stress in aging and high-fat diet-fed aging mice prostate	[90]
Male heterozygous TRAP rats	A diet ad libitum	Anthocyanin-rich fraction from purple rice	0.2 or 1% of hexane insoluble fraction from a purple rice ethanolic extract daily for 10 weeks	Retarded carcinogenesis and castration-resistant cancer growth of prostate through suppression of androgen receptor mediated cell proliferation and metabolism	[60]

C3G: Cyanidin 3-O-glucoside, n.d: not determined, TRAP: transgenic rat for adenocarcinoma of prostate, BPH: benign prostatic hyperplasia.

**Table 3:** Clinical trials evaluating the efficacy of anthocyanins on prostate cancer patients.

Population	Number of Populations + Age Range	Study Type	Diet	Source of Anthocyanin	Anthocyanin Daily Dose	Association between Anthocyanin Intake and CRC Risk	Reference
Patients newly diagnosed with resectable prostate cancer	56 men/average age: 61.6 ± 1.02 years	Interventional	n.d	Nectar of BRB	10 g BRB/day (8 men): 10 g BRB in 1 bottle (total: 320 bottle)	n.a	[93]
				Nectar of BRB	20 g BRB/day (8 men): 20 g BRB in 1 bottle (total: 320 bottle)		
				Confection of BRB	10 g BRB/day (8 men): 10 g BRB in 5 pieces (total: 1600 pieces)		
				Confection of BRB	20 g BRB/day (8 men): 20 g BRB in 10 pieces (total: 3200 pieces)		
Patients receiving image guided intensity modulated radiation therapy to their prostate, prostate and regional lymph nodes or prostate bed were included	41 men/average age: 68 years	Observational	Mostly New Zealand European	cranberry (*Vaccinium macrocarpo*) capsules	1 capsule/day at breakfast during radiation therapy treatment, and for 2 weeks post-treatment (9 weeks for prostate bed, 10 weeks for prostate and prostate nodes)	65% of patients taking cranberry capsules developed cystitis compared to the placebo group (90% patients)	[94]
						30% of patients taking cranberry capsules Developed severe cystitis compared to the placebo group (45% patients) None of them developed urinary tract infections	
Patients receiving external beam radiation therapy to their prostate bed or prostate only and had not received previous pelvic radiation therapy	101 men/average age: 68 years (range 51–85)	Observational	Mostly New Zealand European	cranberry (*Vaccinium macrocarpo*) capsules	2 capsules at breakfast during radiation therapy treatment, For 2 weeks after	Three measurements of cystitis severity: modified RTOG, O’Leary interstitial cystitis scale, RICAS. No significant differences were observed between cranberry treated and placebo groups	[95]

BRB: black raspberry (*Rubus occidentalis*), R TOG: radiation therapy oncology group grading, RICAS: novel radiation induced cystitis assessment scale, na: not analysed, n.d: not determined.

## Data Availability

Not applicable.

## Abbreviations

ACD	anthocyanidin
ACN	anthocyanin
ROS	Reactive oxygen species
HPLC	High-performance liquid chromatography
STAT-3	Signal transducer and activator of transcription 3
CatL	cathepsin L
ER-α	Estrogen receptor-alpha
CDK	Cyclin-dependent kinase
PrEC	Normal prostate epithelial cells
Bcl-2	B-cell lymphoma 2
PSA	Prostate-specific antigen
NF-κB	nuclear factor kappa B
MMP-9	Matrix metallopeptidase 9
DU14	Androgen-sensitive prostate cancer
LnCap	Androgen-independent prostate cancer
CRPC	castrate-resistant prostate cancer
BPH-1	Epithelial cells of benign prostatic hypertrophy
LNCaP	Androgen-dependent tumoral prostatic cells
DU145	Androgen-resistant tumoral prostatic cells
AIF	Apoptosis-inducing factor
C3G	Cyanidin 3-O-glucoside
PGG	petunidin 3-O-[6-O-(4-O-(trans-p-coumaroyl)-α-l-rhamnopyranosyl)-β-d- glucopyranoside]-5-O-[β-d-glucopyranoside]
MMP	matrix metalloproteinase
PC	prostate cancer
pSTAT3	phosphorylated-Signal transducer and activator of transcription-3
